# Sequence analysis of the rifampicin resistance determining region (RRDR) of rpoB gene in multidrug resistance confirmed and newly diagnosed tuberculosis patients of Punjab, Pakistan

**DOI:** 10.1371/journal.pone.0183363

**Published:** 2017-08-17

**Authors:** Salma Hameed, Kartyk Moganeradj, Nasir Mahmood, Timothy D. McHugh, Muhammad Nawaz Chaudhry, Catherine Arnold

**Affiliations:** 1 College of Earth and Environmental Sciences, University of the Punjab, Lahore, Pakistan; 2 Centre for Clinical Microbiology, Division of Infection and Immunity, Royal Free Campus, University College London, London, United Kingdom; 3 Genomic Services and Development Unit, Public Health England, Colindale, United Kingdom; 4 Department of Biochemistry; Department of Human Genetics and Molecular Biology, University of Health Sciences, Lahore, Pakistan; Indian Institute of Science, INDIA

## Abstract

Molecular screening of new patients suspected for TB could help in the effective control of TB in Pakistan as it is a high TB burden country. It will be informative to understand the prevalence of multi drug resistance for a better drug regimen management in this geographical area. The Rifampicin resistance determining region (RRDR) sequencing was used to identify mutations associated with drug resistance in DNA extracts from 130 known multidrug resistant (MDR) cultured strains and compared with mutations observed in DNA extracts directly from 86 sputum samples from consecutive newly diagnosed cases in Lahore, Pakistan. These newly diagnosed samples were positive for smear microscopy, chest X-ray and presumed sensitive to first line drugs. In the known MDR group the most frequent mutations conferring resistance were found in *rpo*B531 (n = 51, 39.2%). In the newly diagnosed tuberculosis group with no history of MDR, mutations in *rpo*B531 were seen in 10 of the samples (11.6%). Collectively, all mutations in the RRDR region studied were observed in 80 (61.5%) of known MDR cases and in 14 (16.3%) of the newly diagnosed cases. Using the RRDR as a surrogate marker for MDR, sequences for the newly diagnosed (presumed sensitive) group indicate much higher levels of MDR than the 3.9% WHO 2015 global estimate and suggests that molecular screening directly from sputum is urgently required to effectively address the detection and treatment gaps to combat MDR in this high burden country.

## Introduction

Pakistan is among the top 20 countries with a high TB and MDR-TB burden [1]. Chest X-ray, acid fast bacilli (AFB) smear microscopy and culture on Lowenstein-Jensen (LJ) media are the conventional methods of investigation for tuberculosis [2] but require additional analysis to define the species of Mycobacteria as well as the mechanism of drug resistance. *M*. *tuberculosis* drug resistance detection using conventional methods is by culture of bacilli on a medium containing antibiotic and can require several weeks. However, with the development of rapid molecular methods it is possible to detect mutations in genes associated with resistance in a much shorter time [3]. Resistance to two first line predominant anti-TB drugs i.e., isoniazid (INH) and rifampicin (RIF), is termed as ‘multidrug resistance tuberculosis’ [4]. Molecular methods for this are diverse and each method has its benefits and drawbacks; for example PCR-RFLP [5] and allele-specific PCR [6]. Several molecular techniques have been evolved to detect the gene mutation related to resistance. These include hybridization methods; single strand polymorphism, DNA sequencing and other PCR based methods [5, 7, 8]. Multiplex Allele Specific (MAS) PCR, a rapid and cost-effective method simultaneously detects INH, RIF and Ethambutol (EMB) resistance associated genetic mutations [9]. PCR technology can provide many advantages over traditional techniques. Many PCR tests can be rapidly performed and interpreted on the same day of submission of samples. A major advantage of PCR over traditional techniques includes the ability to rapidly identify organisms that are difficult to culture and the DNA of interest can be amplified with the DNA from just one cell. The sensitivity of PCR is also its major disadvantage since very small amounts of contaminating DNA (from a different sample) can also be amplified. One major limitation of PCR is that prior information about the target sequence is necessary in order to generate the primers that will allow its selective amplification [10]. Whole Genome Sequencing (WGS) sequences the whole genome rather than specific genes. So, drug resistance prediction from the whole genome sequence is possible using publically available software which rapidly analyses all known gene targets and identifies mutations associated with resistance thus enabling targeted treatment [11] but requires culture and is currently prohibitively expensive for high burden countries. Identification of MDR-TB is a crucial step as treatment of multi drug resistant tuberculosis (MDR-TB) is a considerable challenge. Globally in 2015, an estimated 3.9% (95% confidence interval [CI]: 2.7–5.1%) of new cases and 21% (95% CI: 15–28%) of previously treated cases had MDR/RR-TB [1]. Resistance to rifampicin is the result of mutations in the rifampicin resistance determining region (RRDR) of *rpo*B, particularly mutations at codons 516, 526 and 531. MDR-TB is defined as resistance to rifampicin and isoniazid, the two most effective anti-TB drugs. In December 2010, WHO recommended the use of the GeneXpert MTB/RIF to detect and infer resistance to rifampicin directly from sputum [12]. In May 2016, WHO issued guidance that “people with TB resistant to rifampicin, with or without resistance to other drugs, should be treated with an MDR-TB treatment regimen.” Together with MDR-TB, these are referred to as MDR/RR-TB.

This work was carried out in Pakistan to characterize mutations associated with rifampicin resistance directly from sputum samples from newly diagnosed (ND) patients with no history of drug resistance and identify their key risk factors in this setting. Identifying resistance in presumed resistant samples and inferring resistance profiles directly from sputum may enable a better tailored drug regimen where possible. It will also inform patient management more rapidly and consequently reduce the rate of onward transmission of MDR tuberculosis in high burden countries such as Pakistan.

## Materials and methods

### Sampling

Tuberculosis patients attending the Ghulab Devi Chest Hospital, Lahore, Pakistan in collaboration with University of Health Sciences, Lahore were enrolled in this study over 18 months between 2013 and 2015, based on the following inclusion criteria. For the MDR group, patients diagnosed previously with TB, and with a history of resistance to first line anti-tuberculosis drugs, were included. Sputum samples were taken from this group and culture and Drug Susceptibility Testing (DST) were performed. The second group of patients included freshly diagnosed consecutive cases, presumed drug susceptible with clinical symptoms of TB, positive in sputum smear microscopy AFB, chest X-ray positive and no history of resistance to any first line tuberculosis drugs. Sputum samples were taken from this group and culture was not performed. Not all individuals were included; those with clinical complications in addition to tuberculosis were not included in this study.

### Patient history

The patient’s history was collected using a proforma and included age, gender, area, economic status (earning less than 300 US dollars per month), information of previous anti-tuberculosis therapy, chest x-ray, AFB test and family history of TB. The environmental parameters studied were animal contact, source of drinking water, un-boiled milk use and smoking or drug use.

### GeneXpert testing

GeneXpert testing was carried out only for six samples of the MDR group only due to lack of global funding, according to the manufacturer’s instructions. Newly diagnosed presumed susceptible samples were not tested due to the reason that these samples have no history of drug resistance. This test was performed in order to confirm the samples of MDR group before carrying out DST.

### Initial sputum culturing on drug free LJ medium

For the MDR group, sputum suspension of each patient was made by mixing 0.5ml sputum in equal volume of autoclaved deionized water under aseptic conditions. 0.1ml of sputum sample was spread on LJ medium for *M*. *tuberculosis* culture under strict aseptic conditions. The colonies appeared on the LJ medium after 4–6 weeks of culturing at 37°C.

### Drug susceptibility testing (DST)

Sub culturing of *M*. *tuberculosis* colonies from the MDR group was carried out on LJ medium containing the different first line anti-tuberculosis drugs. The concentrations of drugs added were: rifampicin (40 μg/ml); isoniazid (0.2 μg/ml); ethambutol (2 μg/ml); pyrazinamide (50 μg/ml) andstreptomycin (4 μg/ml). Following a sterility check by incubating the culture bottles at 37°C for one week, the bottles were inoculated with an *M*. *tuberculosis* suspension of the previous culture. After 4–6 weeks incubation at 37°C in incubator, growth on a drug-free control medium was compared with growth on culture media containing each concentration of anti-tuberculosis drug. Any growth of *M*. *tuberculosis* colonies on drug containing LJ media were designated drug resistant while samples where no growth was observed on drug containing LJ media, were declared to be drug susceptible *Mycobacterium tuberculosis*.

### DNA isolation and quantification

Sputum samples from the presumed susceptible group of patients were collected and then DNA extraction was carried out. Both cultures (described above) and sputum samples were extracted using the column based TIANamp genomic DNA isolation kit (TIANGEN Biotech Beijing, China) method. Quantity and quality of the isolated genomic DNA was determined by NanoDrop (Thermo Scientific, USA) using 1μL sample of purified DNA.

### *rpo*B analysis

The fragment containing 81bp Rifampicin Resistant Determining Region (RRDR) of the *rpo*B gene of all strains were sequenced using published primers [13] and analysed in BIOEDIT software using ClustalW alignment parameters (BioEdit version 7.2.5). The PCR was carried out in a total volume of 50μl where 1μl of the DNA was added to the reaction containing 1xPCR reaction buffer, 1.5mM MgCl_2_, 0.2mM dNTPs (Invitrogen, UK), 20μM each of both *rpo*B-RRDR forward (5’- CGATCACACCGCAGACGTTGA) and reverse primers (5’-GGCACGCTCACGTGACAGACC) and 5U recombinant Taq polymerase (Invitrogen, UK). The following PCR conditions were carried out using a Veriti thermocycler (Applied Biosystems, UK): 94°C for 2 min followed by 35 cycles of 94°C for 30 sec, 60°C for 30 sec and 72°C for 1 min. Finally, an extension of 72°C for 10 min was performed before cleaning the products using AmpureXP magnetic beads (Beckman Coulter, UK) according to the manufacturer’s protocol and forward and reverse sequencing performed.

### Ethics and consent

The present research work was approved by the ethical committee of University of the Punjab, Lahore, Pakistan in accordance with the ethical standards of the responsible committee on human experimentation and with the latest (2008) version of Helsinki Declaration of 1975 [14]. The purpose of the study was explained and written consents from the patients or guardians were taken from all patients or from next of their kin, caretakers, or guardians/parents on behalf of all child participants.

## Results

### GeneXpert testing

GeneXpert testing was positive for six of the samples of MDR group and further verified by DST.

### Drug susceptibility testing (DST)

Of the 130 MDR cultures tested for resistance to isoniazid (I), rifampicin (R), ethambutol (E), pyrazinamide (P) and streptomycin (S), 96 were resistant to IREPS, 26 were resistant to IREP, three were resistant to IR, two were resistant to IRES, one was resistant to IRP and two resistant to IRPS. Streptomycin was added so that to kill any other bacterial contamination in the culture in addition to drug sensitive mycobacteria.

### *rpo*B analysis

An overview of the *rpo*B mutations seen in both groups is shown in Table 1. The isolated DNA from all samples quantification was carried out by nanodrop and DNA quantity was found to be in range of 70–90 ng/μl. Of the 130 MDR strains, 80 had mutations in the RRDR region of rpoB (61.3%) (S1 Fig). In order of mutation frequency, 49 strains carried a single mutation at position 531, TCG>TTG/Ser>Leu (65.3%), 8 strains carried a single mutation at position 516, GAC>TAC/Asp>Tyr (10%), 7 strains carried a single mutation at position 516 GAC>GTC/Asp>Val (8.8%), 4 strains carried a single mutation at position 526, CAC>TAC/His>Tyr (5%), 2 strains carried a single mutation at position 516, GAC>GGC/Asp>Gly (2.5%), a further 2 strains carried a single mutation at position 526, CAC>AAC/His>Gly (2.5%) and single strains carried mutations at positions 526, CAC>CCC/His>Pro; 531, TCG>TGG/Ser>Trp; TCG>TGC/Ser>Cys (all at 1.3%). The remaining mutations were as follows: one strain with a deletion of positions 516 and 517; one strain with two mutations, the first at position 516, GAC>GGC/Asp>Ala and the second at position 531, TCG>GCG/Ser>Ala; one strain with two mutations, the first at position 526, CAC>CAG/His>Gln and the second at position 533, CTG>CCG/Leu>Pro; one strain with a two amino acid deletion of 516/7 and two strains with mutations just upstream of the RRDR.

**Table 1:** Mutations seen in rifampicin resistance determining region (RRDR).

	516 (WT = GAC- Asp)			526 (WT = CAC- His)			531 (WT = TCG- Ser)		
SNP	*T*AC	G*T*C	G*G*C	*T*AC	*A*AC	C*C*C	T*T*G	T*G*G	T*GC*
(AA change)	(Tyr)	(Val)	(Gly)	(Tyr)	(Asn)	(Pro)	(Leu)	(Trp)	(Cys)
MDR group (n = 130)	8	7	2	4	2	1	49	1	1
ND group (n = 86)	2	2	0	0	0	0	8	2	0

**Note:** Multidrug resistant group (MDR), Newly diagnosed group (ND).

For the ND group, 14/86 of the extracted DNAs (16.3%) carried mutations associated with MDR status; the remainder showed wild type RRDR sequence. Of the 14, 8 DNAs carried a single mutation at position 531, TCG>TTG/Ser>Leu (57.1%); 2 DNAs carried a single mutation at position 531, TCG>TGG/Ser>Trp (14.3%); 2 DNAs carried a single mutation at position 516, GAC>GTC/Asp>Val (14.3%) and a further 2 DNAs carried a single mutation at position 516, GAC>TAC/Asp>Tyr (14.3%).

## Discussion

Molecular screening of *M*. *tuberculosis*–containing sputum samples for drug resistance, although recommended by the WHO, is expensive and inaccessible to many high incidence areas such as Pakistan. To gain further information about the prevalence of MDR in newly diagnosed patients in this area of Lahore, Pakistan, the RRDR region of the *rpo*B gene from two groups was sequenced; the first group comprised extracted DNA from130 MDR strains from patients diagnosed previously with TB, and with a history of resistance to first line anti-tuberculosis drugs; the second group comprised 86, DNA extracts directly from sputum samples from consecutive newly diagnosed patients, with clinical symptoms of TB, positive in sputum smear microscopy AFB, chest X-ray positive and presumed drug susceptible with no history of resistance to any first line tuberculosis drugs. The most common mutation found in both groups was in line with other studies, i.e. at position 531, TCG>TTG/Ser>Leu, and at 61.3% and 57.1% for MDR and ND groups respectively, at a similar prevalence.

Studies indicate that 96.1% of the rifampicin resistant strains worldwide will have rpoB mutations (so a surrogate marker for MDR) studies [15, 16]. Comparison of the DST results with the RRDR data from the MDR group in this study suggests that only 61.5% of strains carry mutations in this region of the *rpo*B gene so sequencing the RRDR does not correlate with rifampicin resistance as successfully. The reasons for this may be that resistance to rifampicin is conferred by mutations in other parts of the gene or genome or that the DST testing was sub optimal in some way and indicated resistance when none was present, although all patients from this group had a history of resistance to first line anti-tuberculosis drugs. Antimicrobial resistance testing (AST) or Drug resistance testing was established in the 1960s [17] and there is no consensus reference method for MIC determination against which the different methods can be compared to determine common breakpoints.

The main finding of this study however is the high incidence of rifampicin resistance associated mutations, which is often used as a surrogate marker for MDR. At 16.3%, it is considerably higher than the WHO estimate of 3.9% of new cases of multi drug resistant tuberculosis [1]. This single piece of study needs to be justified with the help of future studies to support a high percentage of rif resistant strains. The cost of rpoB sequencing in this study was approximately $10, the cost of a GeneXpert test in this region. The time taken to carry out rpoB sequencing is slightly longer than GeneXpert testing but requires more complex testing and analysis. GeneXpert testing or rpoB sequencing for detection of MDR TB in sputum samples is much faster than waiting for results of culture and DST. The diagnosis and effective treatment for individuals with MDR needs to be tailored and administered quickly by rapid molecular tests and, as a consequence of that, the control of transmission of MDR tuberculosis will be tightened. Only then will MDR tuberculosis infection and transmission be effectively controlled in high incidence areas such as Pakistan, where it is most needed.

## Supporting information

S1 Fig Alignment of RRDR sequences from this research work. (DOC)
