# Supplementary material for: Sequence analysis of the rifampicin resistance determining region (RRDR) of rpoB gene in multidrug resistance confirmed and newly diagnosed tuberculosis patients of Punjab, Pakistan
Source: PLoS One. 2017 Aug 17;12(8):e0183363. doi: 10.1371/journal.pone.0183363 (PMC5560679; doi:10.1371/journal.pone.0183363)
Supplement: S1 Fig — (DOC) [file pone.0183363.s001.doc]

**S1 Fig**

10 20 30 40 50 60 70 80

....|....|....|....|....|....|....|....|....|....|....|....|....|....|....|....|....|..

**H37Rv**  **ATG~GAC~CAG~AAC~AAC~CCG~CTG~TCG~GGG~TTG~ACC~CAC~AAG~CGC~CGA~CTG~TCG~GCG~CTG~GGG~CCC~GGC**

**3S**  **...~...~...~...~...~...~...~...~...~...~...~...~...~...~...~...~...~...~...~...~...~...**

**5S**  **...~...~...~...~...~...~...~...~...~...~...~...~...~...~...~...~...~...~...~...~...~...**

**9S**  **...~...~...~...~...~...~...~...~...~...~...~...~...~...~...~...~.T.~...~...~...~...~...**

**11S**  **...~...~...~...~...~...~...~...~...~...~...~...~...~...~...~...~...~...~...~...~...~...**

**27**  **...~T..~...~...~...~...~...~...~...~...~...~...~...~...~...~...~...~...~...~...~...~...**

**28**  **...~T..~...~...~...~...~...~...~...~...~...~...~...~...~...~...~...~...~...~...~...~...**

**29**  **...~...~...~...~...~...~...~...~...~...~...~...~...~...~...~...~.T.~...~...~...~...~...**

**31**  **...~...~...~...~...~...~...~...~...~...~...~...~...~...~...~...~...~...~...~...~...~...**

**33**  **...~.T.~...~...~...~...~...~...~...~...~...~...~...~...~...~...~...~...~...~...~...~...**

**34**  **...~...~...~...~...~...~...~...~...~...~...~...~...~...~...~...~.T.~...~...~...~...~...**

**35**  **...~...~...~...~...~...~...~...~...~...~...~...~...~...~...~...~.T.~...~...~...~...~...**

**39**  **...~...~...~...~...~...~...~...~...~...~...~...~...~...~...~...~...~...~...~...~...~...**

**43**  **...~...~...~...~...~...~...~...~...~...~...~...~...~...~...~...~.T.~...~...~...~...~...**

**44**  **...~...~...~...~...~...~...~...~...~...~...~...~...~...~...~...~.T.~...~...~...~...~...**

**45**  **...~...~...~...~...~...~...~...~...~...~...~...~...~...~...~...~.G.~...~...~...~...~...**

**46**  **...~...~...~...~...~...~...~...~...~...~...~...~...~...~...~...~.T.~...~...~...~...~...**

**47**  **...~...~...~...~...~...~...~...~...~...~...~...~...~...~...~...~.GC~...~...~...~...~...**

**48**  **...~...~...~...~...~...~...~...~...~...~...~T..~...~...~...~...~...~...~...~...~...~...**

**50**  **...~...~...~...~...~...~...~...~...~...~...~...~...~...~...~...~...~...~...~...~...~...**

**51S**  **...~T..~...~...~...~...~...~...~...~...~...~...~...~...~...~...~...~...~...~...~...~...**

**52S**  **...~...~...~...~...~...~...~...~...~...~...~...~...~...~...~...~.T.~...~...~...~...~...**

**54**  **...~...~...~...~...~...~...~...~...~...~...~...~...~...~...~...~.T.~...~...~...~...~...**

**55**  **...~...~...~...~...~...~...~...~...~...~...~A..~...~...~...~...~...~...~...~...~...~...**

**56**  **...~...~...~...~...~...~...~...~...~...~...~...~...~...~...~...~.T.~...~...~...~...~...**

**57**  **...~.G.~...~...~...~...~...~...~...~...~...~...~...~...~...~...~...~...~...~...~...~...**

**58**  **...~...~...~...~...~...~...~...~...~...~...~...~...~...~...~...~.T.~...~...~...~...~...**

**61**  **...~...~...~...~...~...~...~...~...~...~...~...~...~...~...~...~.T.~...~...~...~...~...**

**62**  **...~...~...~...~...~...~...~...~...~...~...~...~...~...~...~...~.T.~...~...~...~...~...**

**63**  **...~T..~...~...~...~...~...~...~...~...~...~...~...~...~...~...~...~...~...~...~...~...**

**67**  **...~...~...~...~...~...~...~...~...~...~...~...~...~...~...~...~.T.~...~...~...~...~...**

**68**  **...~.T.~...~...~...~...~...~...~...~...~...~...~...~...~...~...~...~...~...~...~...~...**

**72**  **...~...~...~...~...~...~...~...~...~...~...~...~...~...~...~...~.T.~...~...~...~...~...**

**76**  **...~...~...~...~...~...~...~...~...~...~...~...~...~...~...~...~...~...~...~...~...~...**

**77**  **...~...~...~...~...~...~...~...~...~...~...~...~...~...~...~...~.T.~...~...~...~...~...**

**78**  **...~...~...~...~...~...~...~...~...~...~...~...~...~...~...~...~.T.~...~...~...~...~...**

**82**  **...~...~...~...~...~...~...~...~...~...~...~...~...~...~...~...~.T.~...~...~...~...~...**

**87**  **...~...~...~...~...~...~...~...~...~...~...~...~...~...~...~...~...~...~...~...~...~...**

**89**  **...~...~...~...~...~...~...~...~...~...~...~...~...~...~...~...~...~...~...~...~...~...**

**91**  **...~...~...~...~...~...~...~...~...~...~...~...~...~...~...~...~...~...~...~...~...~...**

**99**  **...~...~...~...~...~...~...~...~...~...~...~...~...~...~...~...~.T.~...~...~...~...~...**

**100**  **...~...~...~...~...~...~...~...~...~...~...~.C.~...~...~...~...~...~...~...~...~...~...**

**107**  **...~...~...~...~...~...~...~...~...~...~...~...~...~...~...~...~...~...~...~...~...~...**

**110**  **...~...~...~...~...~...~...~...~...~...~...~...~...~...~...~...~...~...~...~...~...~...**

**111**  **...~...~...~...~...~...~...~...~...~...~...~...~...~...~...~...~...~...~...~...~...~...**

**114**  **...~T..~...~...~...~...~...~...~...~...~...~...~...~...~...~...~...~...~...~...~...~...**

**117**  **...~T..~...~...~...~...~...~...~...~...~...~...~...~...~...~...~...~...~...~...~...~...**

**122**  **...~...~...~...~...~...~...~...~...~...~...~...~...~...~...~...~...~...~...~...~...~...**

**137**  **...~...~...~...~...~...~...~...~...~...~...~...~...~...~...~...~...~...~...~...~...~...**

**161**  **...~...~...~...~...~...~...~...~...~...~...~...~...~...~...~...~.T.~...~...~...~...~...**

**168**  **...~...~...~...~...~...~...~...~...~...~...~...~...~...~...~...~.T.~...~...~...~...~...**

**169**  **...~...~...~...~...~...~...~...~...~...~...~...~...~...~...~...~.T.~...~...~...~...~...**

**170**  **...~.C.~...~...~...~...~...~...~...~...~...~...~...~...~...~...~G..~...~...~...~...~...**

**171**  **...~.T.~...~...~...~...~...~...~...~...~...~...~...~...~...~...~...~...~...~...~...~...**

**172**  **...~...~...~...~...~...~...~...~...~...~...~...~...~...~...~...~.T.~...~...~...~...~...**

**174**  **...~...~...~...~...~...~...~...~...~...~...~...~...~...~...~...~.T.~...~...~...~...~...**

**181**  **...~...~...~...~...~...~...~...~...~...~...~...~...~...~...~...~.T.~...~...~...~...~...**

**187**  **...~...~...~...~...~...~...~...~...~...~...~...~...~...~...~...~...~...~...~...~...~...**

**191**  **...~.T.~...~...~...~...~...~...~...~...~...~...~...~...~...~...~...~...~...~...~...~...**

**192**  **...~...~...~...~...~...~...~...~...~...~...~...~...~...~...~...~...~...~...~...~...~...**

**220**  **...~...~...~...~...~...~...~...~...~...~...~...~...~...~...~...~...~...~...~...~...~...**

**225**  **...~...~...~...~...~...~...~...~...~...~...~...~...~...~...~...~...~...~...~...~...~...**

**241**  **...~...~...~...~...~...~...~...~...~...~...~...~...~...~...~...~.T.~...~...~...~...~...**

**244**  **...~...~...~...~...~...~...~...~...~...~...~...~...~...~...~...~.T.~...~...~...~...~...**

**246**  **...~...~...~...~...~...~...~...~...~...~...~...~...~...~...~...~.T.~...~...~...~...~...**

**256**  **...~...~...~...~...~...~...~...~...~...~...~...~...~...~...~...~.T.~...~...~...~...~...**

**257**  **...~...~...~...~...~...~...~...~...~...~...~...~...~...~...~...~.T.~...~...~...~...~...**

**258**  **...~...~...~...~...~...~...~...~...~...~...~...~...~...~...~...~.T.~...~...~...~...~...**

**259**  **...~...~...~...~...~...~...~...~...~...~...~...~...~...~...~...~.T.~...~...~...~...~...**

**264**  **...~...~...~...~...~...~...~...~...~...~...~...~...~...~...~...~...~...~...~...~...~...**

**268**  **...~...~...~...~...~...~...~...~...~...~...~...~...~...~...~...~...~...~...~...~...~...**

**269**  **...~...~...~...~...~...~...~...~...~...~...~...~...~...~...~...~...~...~...~...~...~...**

**271**  **...~...~...~...~...~...~...~...~...~...~...~...~...~...~...~...~...~...~...~...~...~...**

**273**  **...~...~...~...~...~...~...~...~...~...~...~...~...~...~...~...~...~...~...~...~...~...**

**274**  **...~...~...~...~...~...~...~...~...~...~...~T..~...~...~...~...~...~...~...~...~...~...**

**277**  **...~...~...~...~...~...~...~...~...~...~...~...~...~...~...~...~...~...~...~...~...~...**

**279**  **...~...~...~...~...~...~...~...~...~...~...~...~...~...~...~...~...~...~...~...~...~...**

**280**  **...~...~...~...~...~...~...~...~...~...~...~...~...~...~...~...~...~...~...~...~...~...**

**282**  **...~...~...~...~...~...~...~...~...~...~...~...~...~...~...~...~.T.~...~...~...~...~...**

**284**  **...~...~...~...~...~...~...~...~...~...~...~...~...~...~...~...~...~...~...~...~...~...**

**286**  **...~...~...~...~...~...~...~...~...~...~...~...~...~...~...~...~...~...~...~...~...~...**

**287**  **...~...~...~...~...~...~...~...~...~...~...~...~...~...~...~...~...~...~...~...~...~...**

**288**  **...~...~...~...~...~...~...~...~...~...~...~...~...~...~...~...~...~...~...~...~...~...**

**290**  **...~...~...~...~...~...~...~...~...~...~...~...~...~...~...~...~...~...~...~...~...~...**

**291**  **...~...~...~...~...~...~...~...~...~...~...~...~...~...~...~...~...~...~...~...~...~...**

**270**  **...~...~...~...~...~...~...~...~...~...~...~...~...~...~...~...~...~...~...~...~...~...**

**14S**  **...~...~...~...~...~...~...~...~...~...~...~...~...~...~...~...~.T.~...~...~...~...~...**

**15S**  **...~...~...~...~...~...~...~...~...~...~...~...~...~...~...~...~...~...~...~...~...~...**

**18S**  **...~...~...~...~...~...~...~...~...~...~...~...~...~...~...~...~...~...~...~...~...~...**

**23S**  **...~...~...~...~...~...~...~...~...~...~...~...~...~...~...~...~...~...~...~...~...~...**

**30**  **...~...~...~...~...~...~...~...~...~...~...~...~...~...~...~...~...~...~...~...~...~...**

**32**  **...~T..~...~...~...~...~...~...~...~...~...~...~...~...~...~...~...~...~...~...~...~...**

**37**  **...~...~...~...~...~...~...~...~...~...~...~...~...~...~...~...~...~...~...~...~...~...**

**37**  **...~...~...~...~...~...~...~...~...~...~...~...~...~...~...~...~.T.~...~...~...~...~...**

**40**  **...~...~...~...~...~...~...~...~...~...~...~...~...~...~...~...~.T.~...~...~...~...~...**

**41**  **...~...~...~...~...~...~...~...~...~...~...~...~...~...~...~...~.T.~...~...~...~...~...**

**42**  **...~...~...~...~...~...~...~...~...~...~...~...~...~...~...~...~.T.~...~...~...~...~...**

**59**  **...~...~...~...~...~...~...~...~...~...~...~...~...~...~...~...~...~...~...~...~...~...**

**64**  **...~.T.~...~...~...~...~...~...~...~...~...~...~...~...~...~...~...~...~...~...~...~...**

**66**  **...~.T.~...~...~...~...~...~...~...~...~...~...~...~...~...~...~...~...~...~...~...~...**

**69**  **...~...~...~...~...~...~...~...~...~...~...~...~...~...~...~...~...~...~...~...~...~...**

**70**  **...~...~...~...~...~...~...~...~...~...~...~...~...~...~...~...~.T.~...~...~...~...~...**

**74**  **...~...~...~...~...~...~...~...~...~...~...~...~...~...~...~...~...~...~...~...~...~...**

**75**  **...~...~...~...~...~...~...~...~...~...~...~...~...~...~...~...~...~...~...~...~...~...**

**80**  **...~...~...~...~...~...~...~...~...~...~...~...~...~...~...~...~...~...~...~...~...~...**

**88**  **...~...~...~...~...~...~...~...~...~...~...~...~...~...~...~...~...~...~...~...~...~...**

**90**  **...~...~...~...~...~...~...~...~...~...~...~...~...~...~...~...~.T.~...~...~...~...~...**

**92**  **...~...~...~...~...~...~...~...~...~...~...~T..~...~...~...~...~...~...~...~...~...~...**

**96**  **...~...~...~...~...~...~...~...~...~...~...~...~...~...~...~...~.T.~...~...~...~...~...**

**101**  **...~...~...~...~...~...~...~...~...~...~...~...~...~...~...~...~.T.~...~...~...~...~...**

**104**  **...~.G.~...~...~...~...~...~...~...~...~...~...~...~...~...~...~...~...~...~...~...~...**

**105**  **...~...~...~...~...~...~...~...~...~...~...~A..~...~...~...~...~...~...~...~...~...~...**

**113**  **...~...~...~...~...~...~...~...~...~...~...~...~...~...~...~...~...~...~...~...~...~...**

**136**  **...~~~~~~~~~...~...~...~...~...~...~...~...~...~...~...~...~...~...~...~...~...~...~...**

**163**  **...~...~...~...~...~...~...~...~...~...~...~...~...~...~...~...~.T.~...~...~...~...~...**

**165**  **...~...~...~...~...~...~...~...~...~...~...~...~...~...~...~...~.T.~...~...~...~...~...**

**166**  **...~...~...~...~...~...~...~...~...~...~...~...~...~...~...~...~...~...~...~...~...~...**

**167**  **...~...~...~...~...~...~...~...~...~...~...~...~...~...~...~...~.T.~...~...~...~...~...**

**176**  **...~...~...~...~...~...~...~...~...~...~...~...~...~...~...~...~.T.~...~...~...~...~...**

**179**  **...~...~...~...~...~...~...~...~...~...~...~...~...~...~...~...~...~...~...~...~...~...**

**182**  **...~T..~...~...~...~...~...~...~...~...~...~...~...~...~...~...~...~...~...~...~...~...**

**189**  **...~...~...~...~...~...~...~...~...~...~...~...~...~...~...~...~...~...~...~...~...~...**

**248**  **...~.T.~...~...~...~...~...~...~...~...~...~...~...~...~...~...~...~...~...~...~...~...**

**249**  **...~...~...~...~...~...~...~...~...~...~...~..G~...~...~...~...~...~...~.C.~...~...~...**

**257**  **...~...~...~...~...~...~...~...~...~...~...~...~...~...~...~...~.T.~...~...~...~...~...**

**261**  **...~...~...~...~...~...~...~...~...~...~...~...~...~...~...~...~...~...~...~...~...~...**

**262**  **...~...~...~...~...~...~...~...~...~...~...~T..~...~...~...~...~...~...~...~...~...~...**

**281**  **...~...~...~...~...~...~...~...~...~...~...~...~...~...~...~...~...~...~...~...~...~...**

**285**  **...~...~...~...~...~...~...~...~...~...~...~...~...~...~...~...~...~...~...~...~...~...**

**289**  **...~...~...~...~...~...~...~...~...~...~...~...~...~...~...~...~...~...~...~...~...~...**

**3080**  **...~...~...~...~...~...~...~...~...~...~...~...~...~...~...~...~.T.~...~...~...~...~...**

**18**  **...~...~...~...~...~...~...~...~...~...~...~...~...~...~...~...~.T.~...~...~...~...~...**

**21S**  **...~...~...~...~...~...~...~...~...~...~...~...~...~...~...~...~...~...~...~...~...~...**

**24S**  **...~...~...~...~...~...~...~...~...~...~...~...~...~...~...~...~...~...~...~...~...~...**

**60**  **...~...~...~...~...~...~...~...~...~...~...~...~...~...~...~...~.T.~...~...~...~...~...**

**81**  **...~T..~...~...~...~...~...~...~...~...~...~...~...~...~...~...~...~...~...~...~...~...**

**83**  **...~.T.~...~...~...~...~...~...~...~...~...~...~...~...~...~...~...~...~...~...~...~...**

**84**  **...~...~...~...~...~...~...~...~...~...~...~...~...~...~...~...~...~...~...~...~...~...**

**85**  **...~...~...~...~...~...~...~...~...~...~...~...~...~...~...~...~...~...~...~...~...~...**

**93**  **...~...~...~...~...~...~...~...~...~...~...~...~...~...~...~...~...~...~...~...~...~...**

**103**  **...~...~...~...~...~...~...~...~...~...~...~...~...~...~...~...~.T.~...~...~...~...~...**

**119**  **...~...~...~...~...~...~...~...~...~...~...~...~...~...~...~...~...~...~...~...~...~...**

**120**  **...~...~...~...~...~...~...~...~...~...~...~...~...~...~...~...~...~...~...~...~...~...**

**121**  **...~...~...~...~...~...~...~...~...~...~...~...~...~...~...~...~...~...~...~...~...~...**

**123**  **...~...~...~...~...~...~...~...~...~...~...~...~...~...~...~...~...~...~...~...~...~...**

**124**  **...~...~...~...~...~...~...~...~...~...~...~...~...~...~...~...~...~...~...~...~...~...**

**125**  **...~...~...~...~...~...~...~...~...~...~...~...~...~...~...~...~...~...~...~...~...~...**

**127**  **...~...~...~...~...~...~...~...~...~...~...~...~...~...~...~...~...~...~...~...~...~...**

**129**  **...~...~...~...~...~...~...~...~...~...~...~...~...~...~...~...~...~...~...~...~...~...**

**130**  **...~...~...~...~...~...~...~...~...~...~...~...~...~...~...~...~...~...~...~...~...~...**

**131**  **...~...~...~...~...~...~...~...~...~...~...~...~...~...~...~...~...~...~...~...~...~...**

**132**  **...~...~...~...~...~...~...~...~...~...~...~...~...~...~...~...~...~...~...~...~...~...**

**133**  **...~...~...~...~...~...~...~...~...~...~...~...~...~...~...~...~...~...~...~...~...~...**

**134**  **...~...~...~...~...~...~...~...~...~...~...~...~...~...~...~...~...~...~...~...~...~...**

**138**  **...~...~...~...~...~...~...~...~...~...~...~...~...~...~...~...~...~...~...~...~...~...**

**139**  **...~...~...~...~...~...~...~...~...~...~...~...~...~...~...~...~...~...~...~...~...~...**

**140**  **...~...~...~...~...~...~...~...~...~...~...~...~...~...~...~...~...~...~...~...~...~...**

**142**  **...~...~...~...~...~...~...~...~...~...~...~...~...~...~...~...~...~...~...~...~...~...**

**143**  **...~...~...~...~...~...~...~...~...~...~...~...~...~...~...~...~...~...~...~...~...~...**

**145**  **...~...~...~...~...~...~...~...~...~...~...~...~...~...~...~...~...~...~...~...~...~...**

**147**  **...~...~...~...~...~...~...~...~...~...~...~...~...~...~...~...~...~...~...~...~...~...**

**148**  **...~...~...~...~...~...~...~...~...~...~...~...~...~...~...~...~...~...~...~...~...~...**

**149**  **...~...~...~...~...~...~...~...~...~...~...~...~...~...~...~...~...~...~...~...~...~...**

**150**  **...~...~...~...~...~...~...~...~...~...~...~...~...~...~...~...~...~...~...~...~...~...**

**151**  **...~...~...~...~...~...~...~...~...~...~...~...~...~...~...~...~...~...~...~...~...~...**

**152**  **...~...~...~...~...~...~...~...~...~...~...~...~...~...~...~...~...~...~...~...~...~...**

**153**  **...~...~...~...~...~...~...~...~...~...~...~...~...~...~...~...~...~...~...~...~...~...**

**155**  **...~...~...~...~...~...~...~...~...~...~...~...~...~...~...~...~.T.~...~...~...~...~...**

**156**  **...~...~...~...~...~...~...~...~...~...~...~...~...~...~...~...~.T.~...~...~...~...~...**

**157**  **...~...~...~...~...~...~...~...~...~...~...~...~...~...~...~...~...~...~...~...~...~...**

**158**  **...~...~...~...~...~...~...~...~...~...~...~...~...~...~...~...~...~...~...~...~...~...**

**159**  **...~...~...~...~...~...~...~...~...~...~...~...~...~...~...~...~...~...~...~...~...~...**

**164**  **...~...~...~...~...~...~...~...~...~...~...~...~...~...~...~...~.T.~...~...~...~...~...**

**175**  **...~...~...~...~...~...~...~...~...~...~...~...~...~...~...~...~...~...~...~...~...~...**

**178**  **...~...~...~...~...~...~...~...~...~...~...~...~...~...~...~...~...~...~...~...~...~...**

**183**  **...~...~...~...~...~...~...~...~...~...~...~...~...~...~...~...~.G.~...~...~...~...~...**

**184**  **...~...~...~...~...~...~...~...~...~...~...~...~...~...~...~...~.T.~...~...~...~...~...**

**185**  **...~...~...~...~...~...~...~...~...~...~...~...~...~...~...~...~...~...~...~...~...~...**

**186**  **...~...~...~...~...~...~...~...~...~...~...~...~...~...~...~...~.T.~...~...~...~...~...**

**188**  **...~...~...~...~...~...~...~...~...~...~...~...~...~...~...~...~...~...~...~...~...~...**

**193**  **...~...~...~...~...~...~...~...~...~...~...~...~...~...~...~...~...~...~...~...~...~...**

**194**  **...~...~...~...~...~...~...~...~...~...~...~...~...~...~...~...~...~...~...~...~...~...**

**195**  **...~...~...~...~...~...~...~...~...~...~...~...~...~...~...~...~...~...~...~...~...~...**

**196**  **...~...~...~...~...~...~...~...~...~...~...~...~...~...~...~...~...~...~...~...~...~...**

**197**  **...~...~...~...~...~...~...~...~...~...~...~...~...~...~...~...~...~...~...~...~...~...**

**198**  **...~...~...~...~...~...~...~...~...~...~...~...~...~...~...~...~...~...~...~...~...~...**

**200**  **...~.T.~...~...~...~...~...~...~...~...~...~...~...~...~...~...~...~...~...~...~...~...**

**201**  **...~...~...~...~...~...~...~...~...~...~...~...~...~...~...~...~...~...~...~...~...~...**

**203**  **...~...~...~...~...~...~...~...~...~...~...~...~...~...~...~...~...~...~...~...~...~...**

**204**  **...~...~...~...~...~...~...~...~...~...~...~...~...~...~...~...~...~...~...~...~...~...**

**205**  **...~...~...~...~...~...~...~...~...~...~...~...~...~...~...~...~...~...~...~...~...~...**

**206**  **...~...~...~...~...~...~...~...~...~...~...~...~...~...~...~...~...~...~...~...~...~...**

**207**  **...~...~...~...~...~...~...~...~...~...~...~...~...~...~...~...~...~...~...~...~...~...**

**208**  **...~...~...~...~...~...~...~...~...~...~...~...~...~...~...~...~...~...~...~...~...~...**

**209**  **...~...~...~...~...~...~...~...~...~...~...~...~...~...~...~...~...~...~...~...~...~...**

**210**  **...~...~...~...~...~...~...~...~...~...~...~...~...~...~...~...~...~...~...~...~...~...**

**211**  **...~...~...~...~...~...~...~...~...~...~...~...~...~...~...~...~...~...~...~...~...~...**

**212**  **...~...~...~...~...~...~...~...~...~...~...~...~...~...~...~...~...~...~...~...~...~...**

**214**  **...~...~...~...~...~...~...~...~...~...~...~...~...~...~...~...~...~...~...~...~...~...**

**216**  **...~...~...~...~...~...~...~...~...~...~...~...~...~...~...~...~...~...~...~...~...~...**

**217**  **...~...~...~...~...~...~...~...~...~...~...~...~...~...~...~...~...~...~...~...~...~...**

**218**  **...~...~...~...~...~...~...~...~...~...~...~...~...~...~...~...~...~...~...~...~...~...**

**222**  **...~...~...~...~...~...~...~...~...~...~...~...~...~...~...~...~...~...~...~...~...~...**

**223**  **...~...~...~...~...~...~...~...~...~...~...~...~...~...~...~...~...~...~...~...~...~...**

**226**  **...~...~...~...~...~...~...~...~...~...~...~...~...~...~...~...~...~...~...~...~...~...**

**230**  **...~...~...~...~...~...~...~...~...~...~...~...~...~...~...~...~...~...~...~...~...~...**

**231**  **...~...~...~...~...~...~...~...~...~...~...~...~...~...~...~...~.G.~...~...~...~...~...**

**232**  **...~...~...~...~...~...~...~...~...~...~...~...~...~...~...~...~...~...~...~...~...~...**

**233**  **...~...~...~...~...~...~...~...~...~...~...~...~...~...~...~...~...~...~...~...~...~...**

**234**  **...~...~...~...~...~...~...~...~...~...~...~...~...~...~...~...~...~...~...~...~...~...**

**235**  **...~...~...~...~...~...~...~...~...~...~...~...~...~...~...~...~...~...~...~...~...~...**

**237**  **...~...~...~...~...~...~...~...~...~...~...~...~...~...~...~...~...~...~...~...~...~...**

**239**  **...~...~...~...~...~...~...~...~...~...~...~...~...~...~...~...~...~...~...~...~...~...**

**247**  **...~...~...~...~...~...~...~...~...~...~...~...~...~...~...~...~.T.~...~...~...~...~...**

**255**  **...~...~...~...~...~...~...~...~...~...~...~...~...~...~...~...~...~...~...~...~...~...**
